# Supplementary material for: The eATP/P2×7R Axis Drives Quantum Dot‐Nanoparticle Induced Neutrophil Recruitment in the Pulmonary Microcirculation
Source: Adv Sci (Weinh). 2024 Oct 4;11(45):2404661. doi: 10.1002/advs.202404661 (PMC11615809; doi:10.1002/advs.202404661)
Supplement: Supplementary file 1 — Supporting Information [file ADVS-11-2404661-s005.docx]

**Supplemental figures and videos**


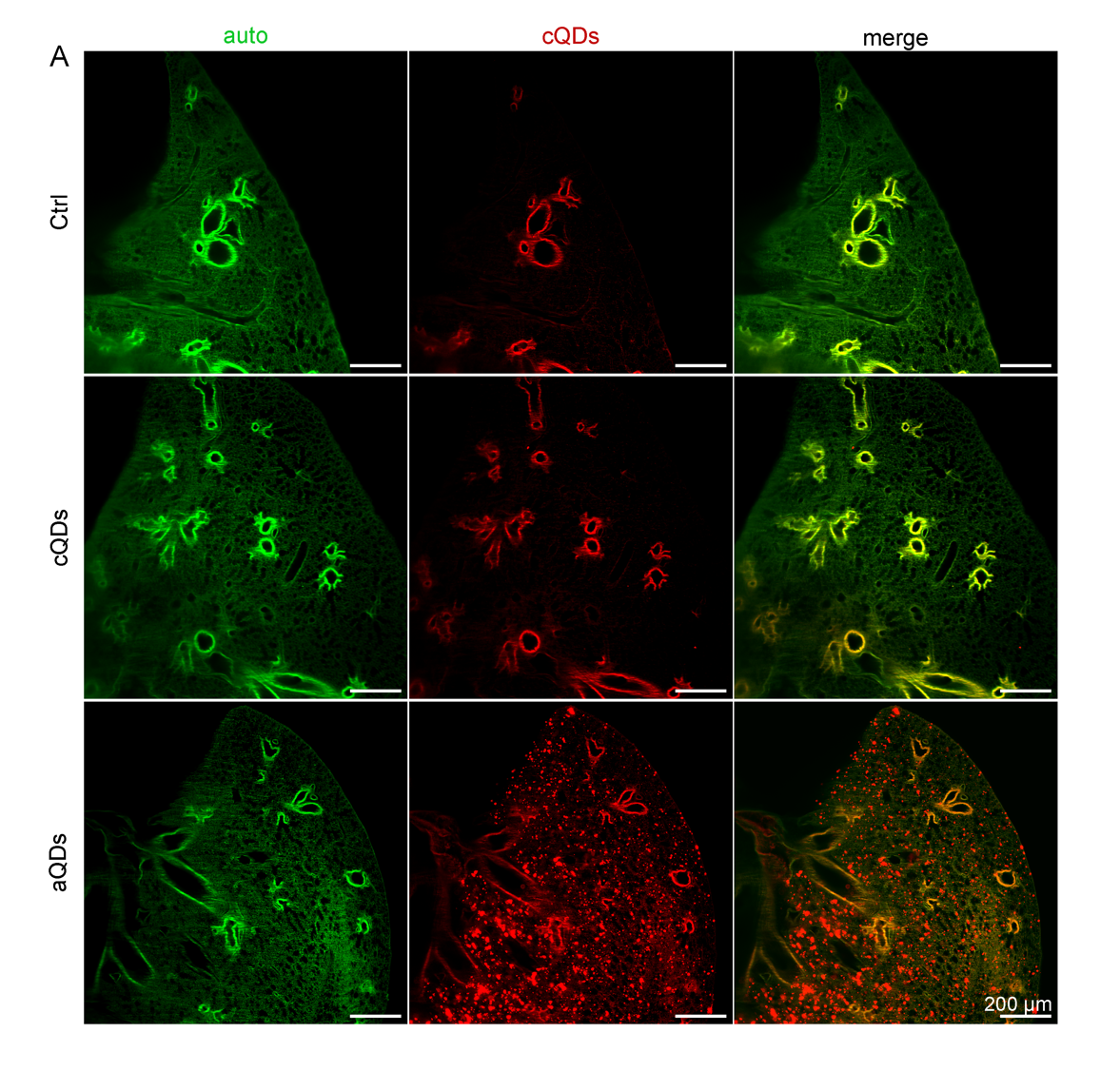


**Supplemental figure 1 and video S1, S2, S3: QDs distribution in whole lungs**

After intravenous application of vehicle (video S1), cQDs (video S2), or aQDs (video S3) for 1 hour, lung tissues were optically cleared and imaged in 3D. The lung structure is represented in green, while QDs are visualized in a bright red color, providing an overall presentation of their distribution within the lung.


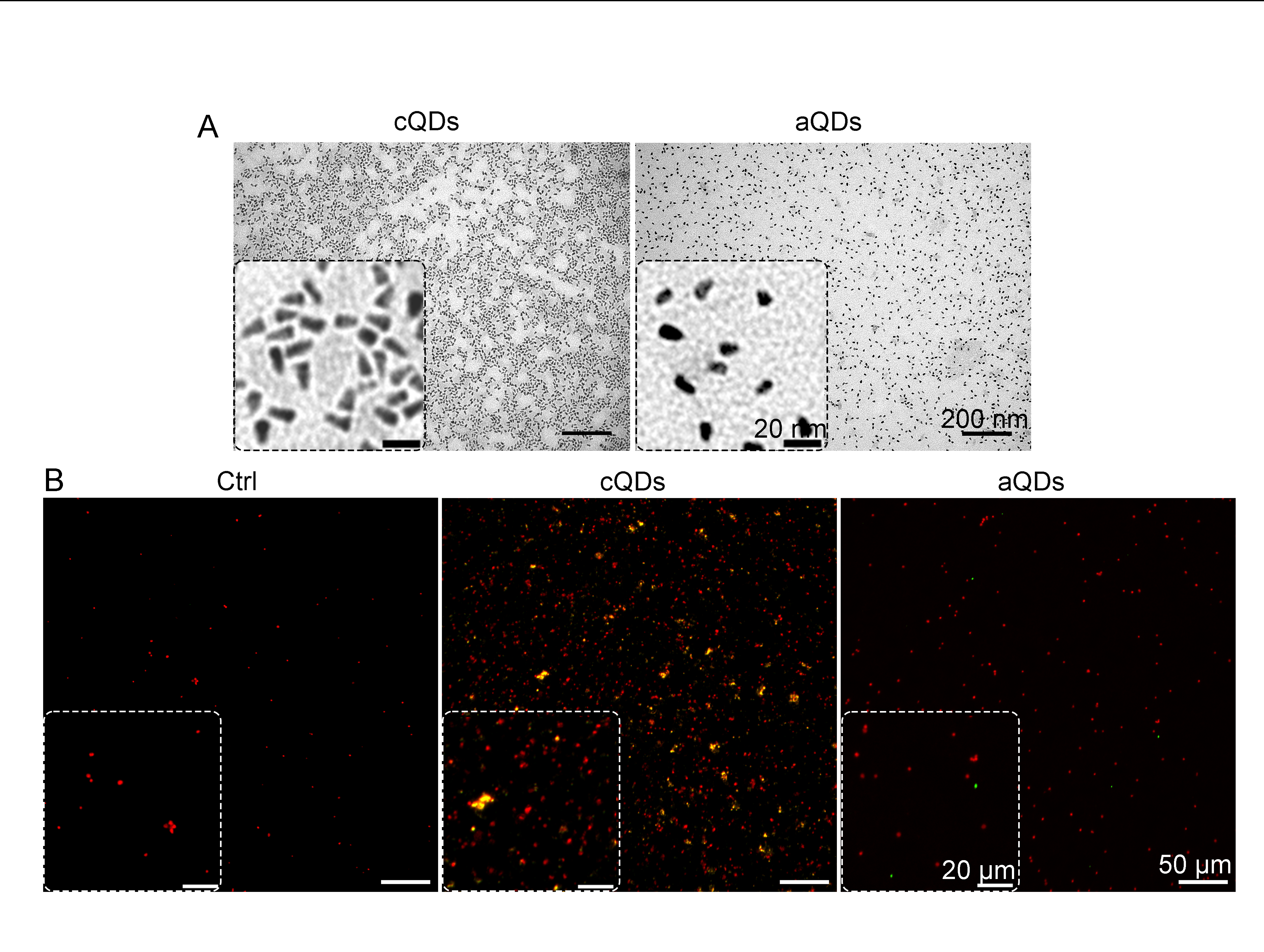


**Supplemental figure 2: QDs are monodisperse prior to injection and do not form aggregates when incubated with blood.**

(A) TEM of cQDs and aQDs diluted in H_2_O. (Scale bars: 20/200 nm). (B) Incubation of cQDs and aQDs with mouse blood. The blood was freshly collected from mice, stained with an anti-platelet antibody, to visualize blood components (DyLight 649 anti-GPIbβ mAb, emfret Analytics, Germany), and incubated with cQDs or aQDs (8 nm) for 30 min. QDs are green and platelets are red. (Scale bars: 20/50 μm).


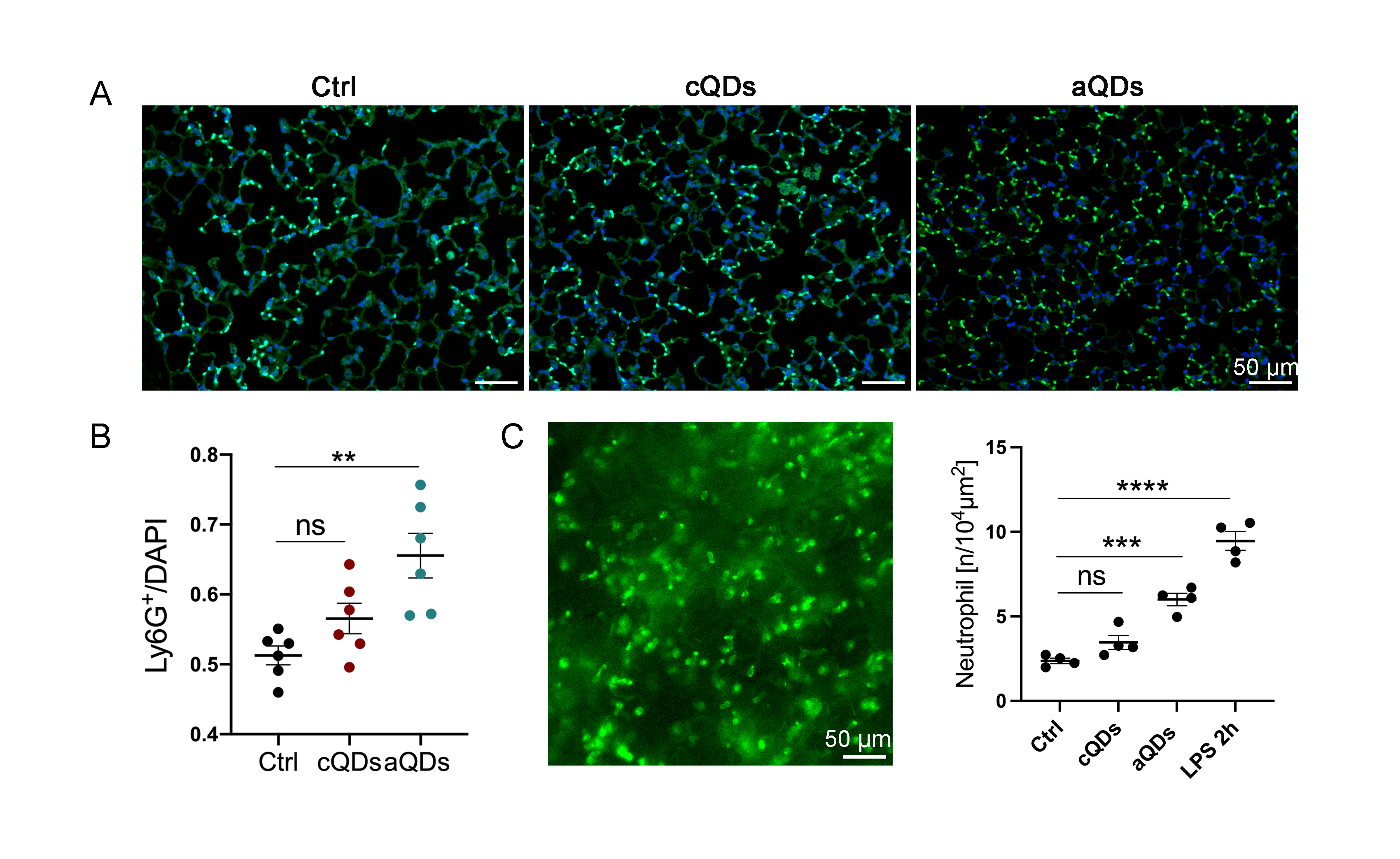


**Supplemental figure 3: Histology of lung slices indicates increased neutrophil numbers after aQDs application and L-IVM comparison of aQD to endotoxin (LPS) induced inflammation.**

(A) Neutrophils were stained with Alexa488-labeled anti-Ly6G antibody in lung slices and are depicted in white. (B) Quantification of neutrophils is shown. n = 2 tissue slices from 3 mice/group, mean ± SEM, analyzed using Student’s t-test; ** indicates P ≤ 0.01. (C) (Left) Representative L-IVM image of neutrophil accumulations obtained after 2h of LPS instillation into the lung. (Right) Quantification of LPS induced neutrophil recruitment to those induced by cQDs and aQDs 60 min after iv injection. Control, cQDs, and aQD neutrophil counts at 60 min are taken from Fig. 2 B, timepoint 60min. n = 4 mice/group, mean ± SEM, One-way ANOVA. *** indicates P ≤ 0.001, and **** indicates P ≤ 0.0001. Scale bar: 50 μm.


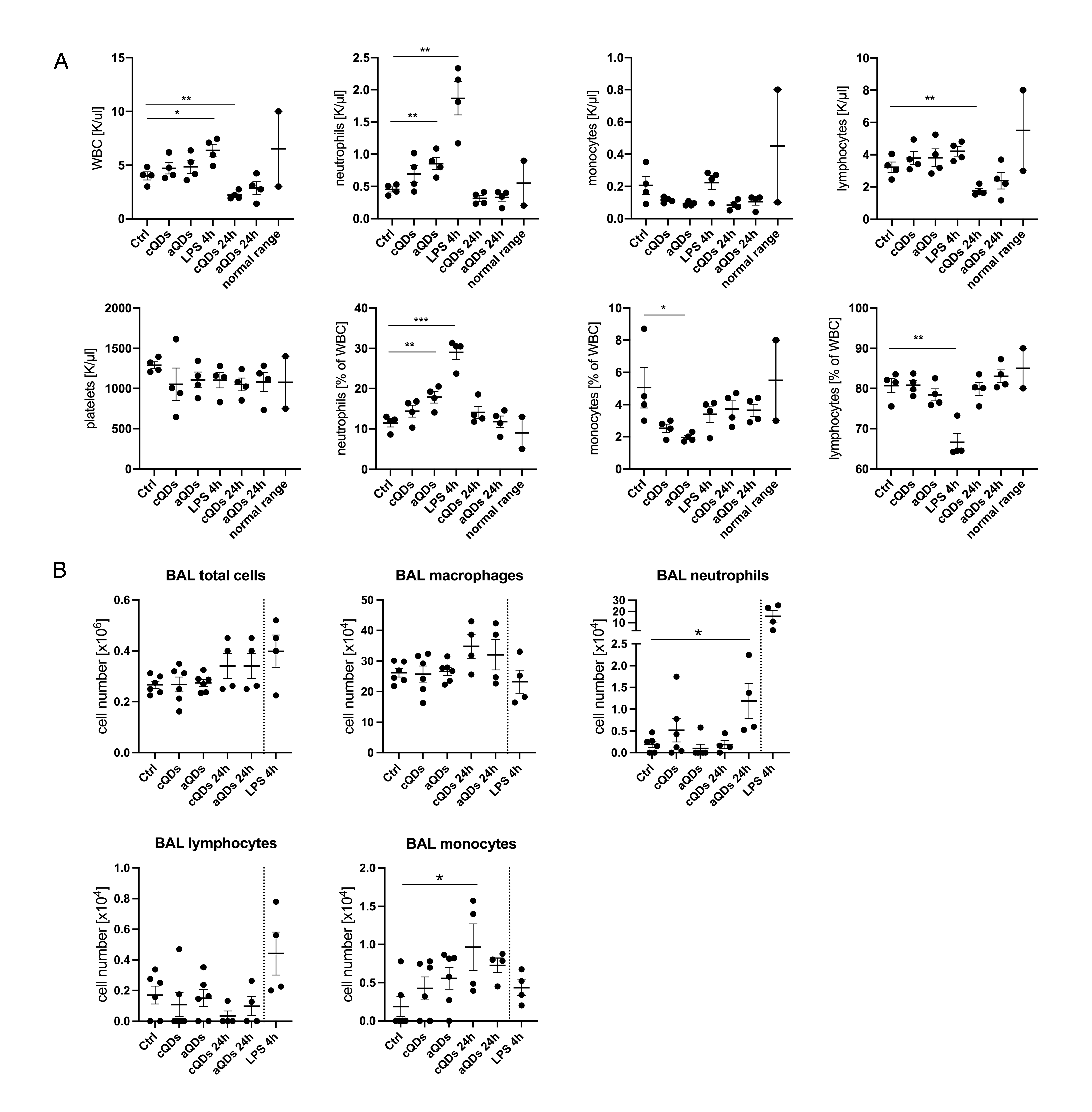


**Supplemental figure 4: Changes in systemic blood parameters and BAL cell counts following QDs application.**

(A) Systemic level and percentage of immune cells 1-hour or 24-hour after QDs application. (mean ± SEM, n = 4 mice/group, Student’s t-test). (B) Quantification of total BAL cells, macrophages, neutrophils, lymphocytes, and monocytes after 1-hour or 24-hour QDs application, and 4h after LPS induced lung inflammation (positive control) is shown. May Grunwald-Giemsa stained cytospin samples of BAL cells were analyzed. mean ± SEM, n = 4-6 mice/group, Student’s t-test. * Indicates P ≤ 0.05, ** indicates P ≤ 0.01, *** indicates P ≤ 0.001, and **** indicates P ≤ 0.0001.


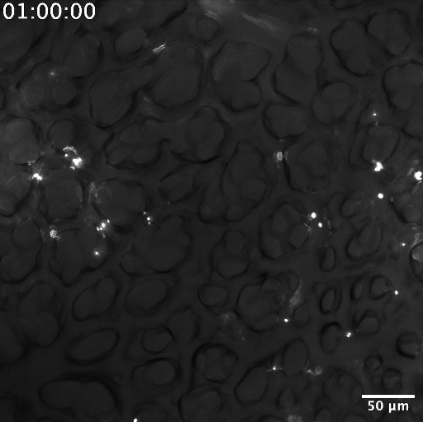


**Supplemental video S5 (still): Blood flow velocity in pulmonary microvessels**

Video shows blood flow depicted by bead trajectories in the lung microcirculation. The sequential images reveal blood flow direction in blood vessels presented by individual microbead trajectories, depicted as different colored lines.


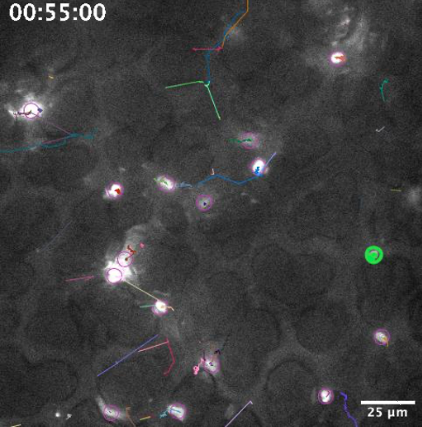


**Supplemental video S6 (still): Trajectories of neutrophil movements in healthy mice via L-IVM**

Neutrophil dynamics were recorded every 5 seconds over a period of 10 min by L-IVM. The images were analyzed, and the movement trajectories of neutrophils were automatically generated by plugin “Trackmate” of Fiji software. The video displays representative neutrophil trajectories during the period of 55-65 min under healthy conditions. Each trajectory is represented by a different color, indicating tracks of individual neutrophils. (Scale bar: 25 μm).


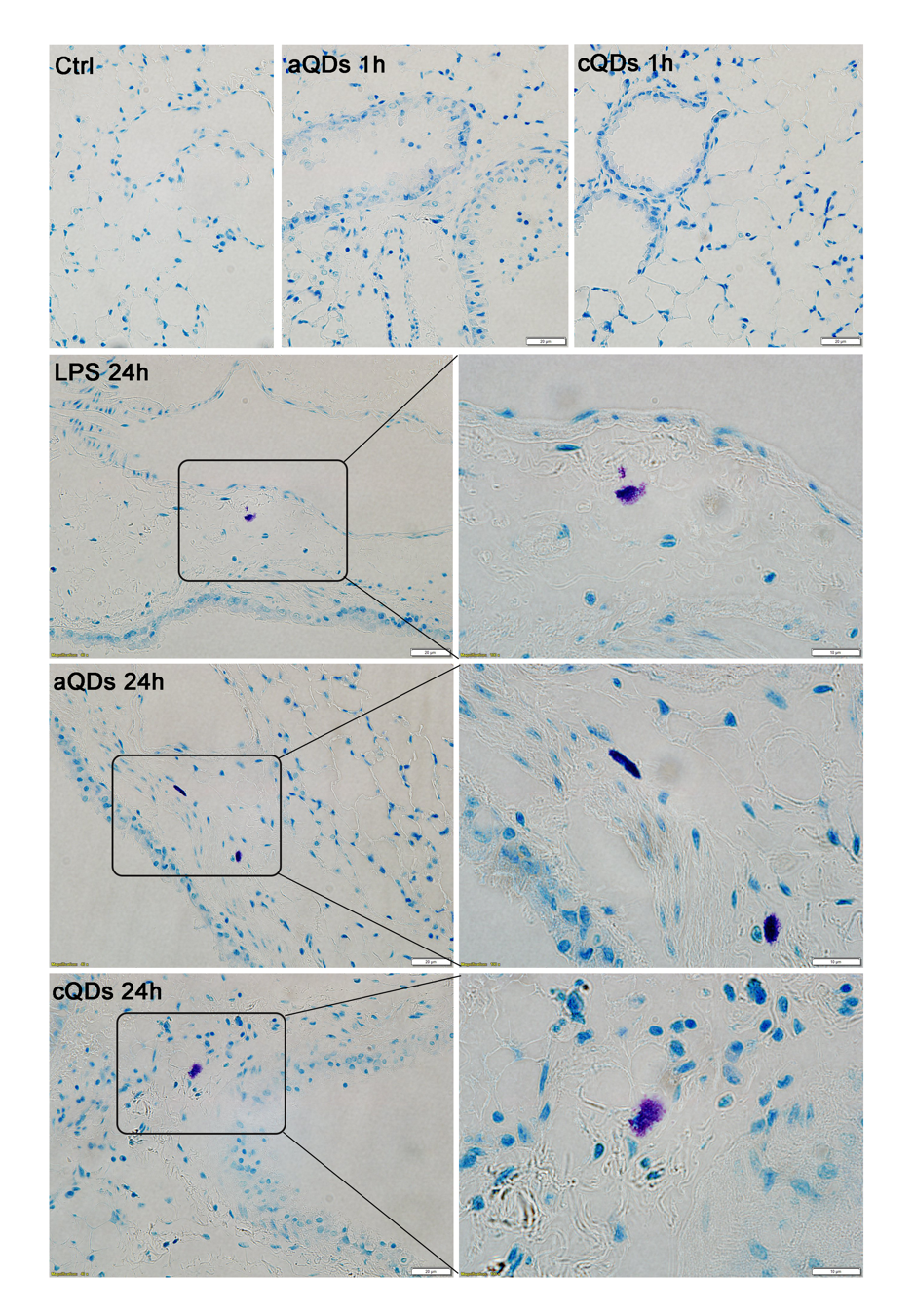


**Supplemental figure 7: Metachromatic granule staining in mast cells after aQDs or cQDs exposure, LPS treatment, and under control conditions**

The images display metachromatic granule staining in mast cells, visible as violet against the blue background of surrounding tissues, obtained after tolouidin staining, after 1 h and 24 h aQDs or cQDs exposure, 24 h LPS instillation (0.1 μg/mouse), or vehicle control. (Scale bar: 10/20 µm).


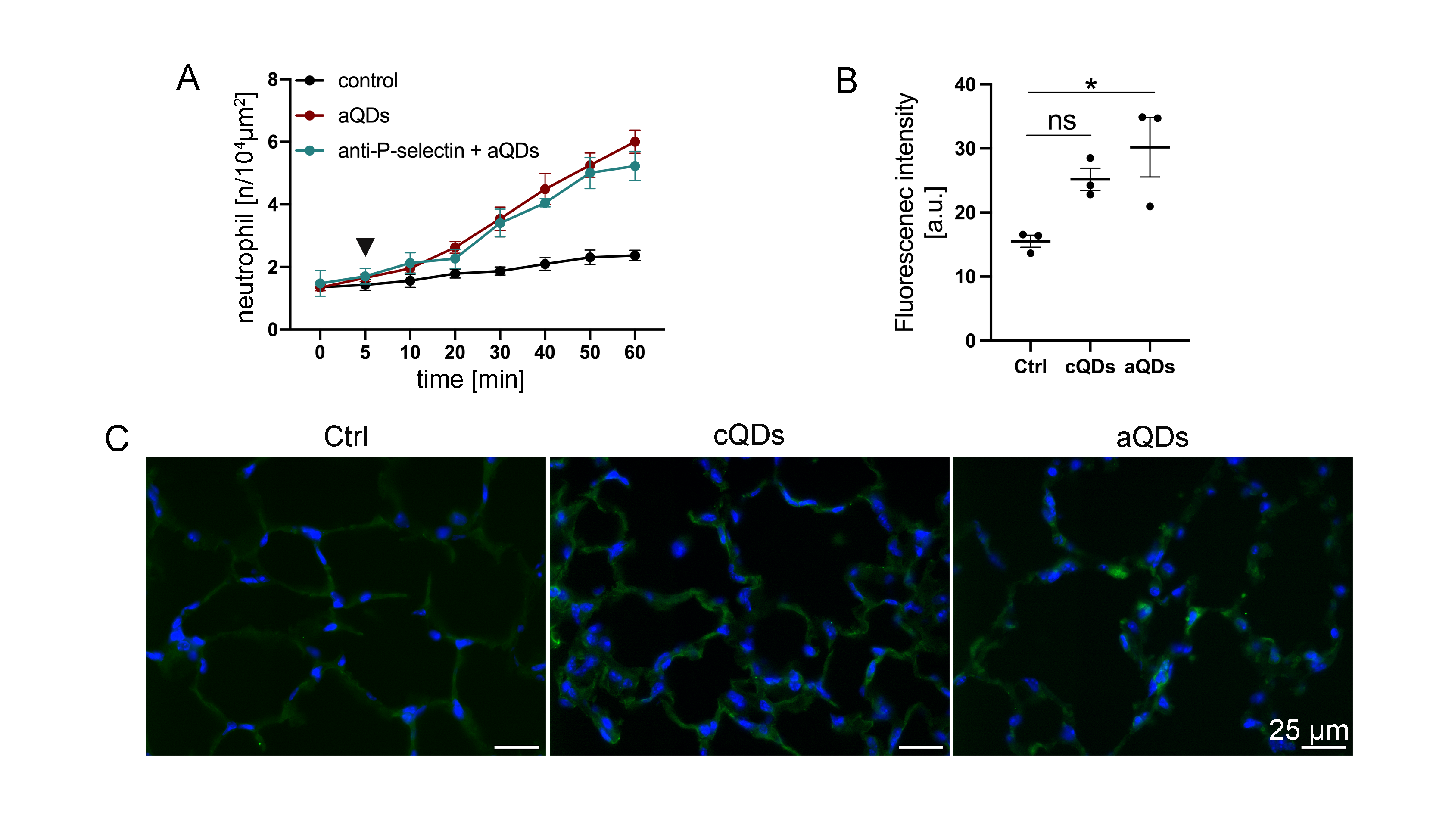


**Supplemental figure 8: Inhibiting P-selectins did not alter neutrophil recruitment dynamics, whereas ICAM-1 expression was increased in response to aQDs.**

(A) Mice were intravenously pre-treated with anti-P-selectin mAbs for 30 minutes before aQDs application, compared to aQDs-only application and control groups. Neutrophil numbers were quantified over time. Black arrow indicates aQDs injection at t = 5 min; mean ± SEM, n = 4 mice/group. Control and aQD neutrophil counts same data as in Fig. 2B. (B) Quantification of ICAM-1fluorescence intensities in lung histology samples from control, cQDs, and aQDs- treated mice after 1h, (C) corresponding representative lung slices stained with rat anti-ICAM1 antibody (green) and DAPI (blue). Scale bars: 25 μm, mean ± SEM, n = 3 mice/group, * indicates P ≤ 0.05.


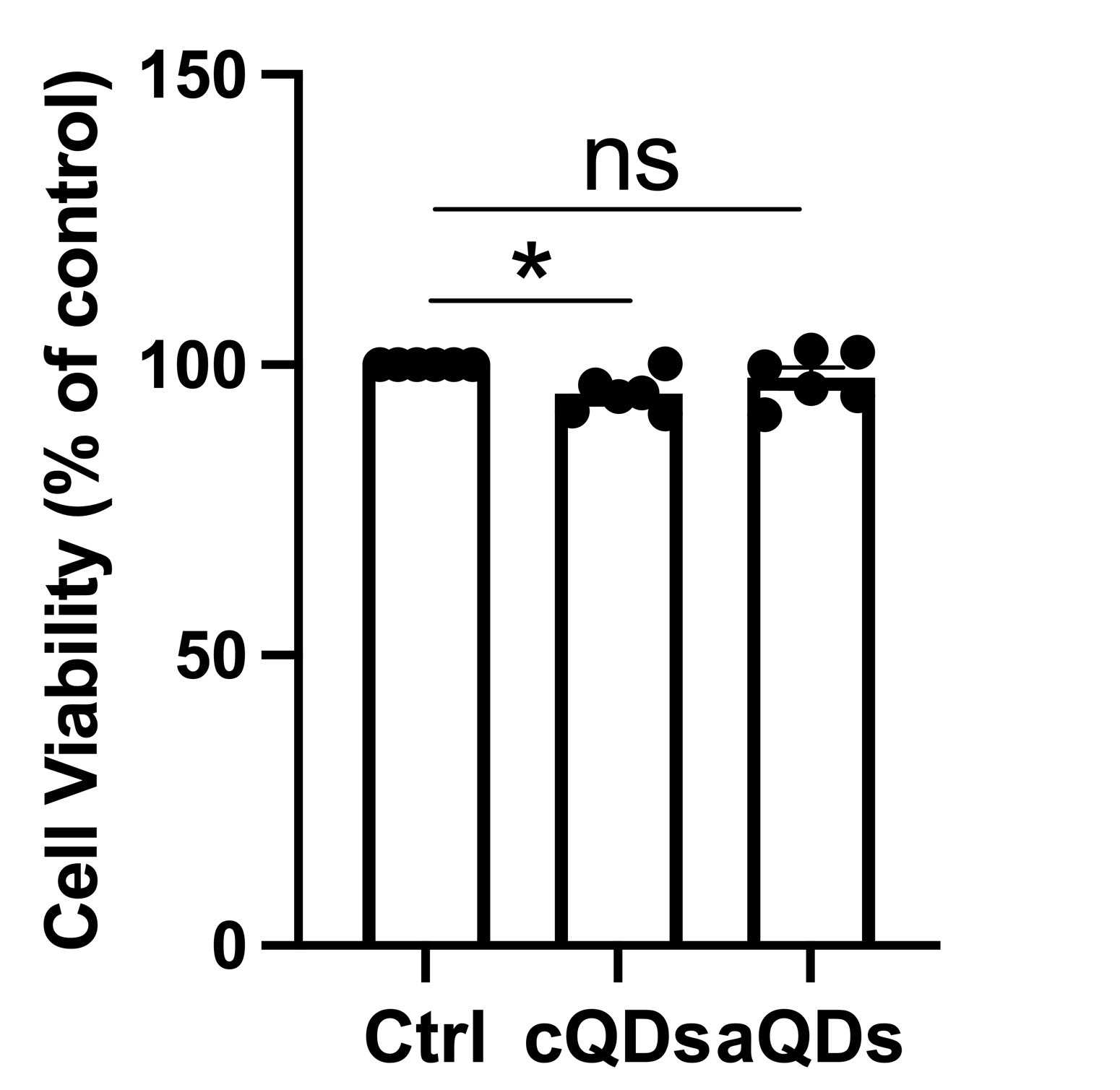


**Supplemental figure 9: aQDs do not affect MHS cell viability.**

MHS cells were exposed to cQDs or aQDs (8 nM) or vehicle (control) for 2h. Thereafter cell viability was determined by WST-1 assay. Data is shown as mean ± SEM, n = 6 independent experiments. One-way ANOVA test, * indicates P ≤ 0.05.

**Supplemental Table S1: List of in vivo fluorescent dyes, blocking antibodies, and antagonists.**

| Name | Clone | Company | Dose |
| --- | --- | --- | --- |
| A438079 hydrochloride (Competitive P2X_7_ antagonist) | ≥ 98 %  (HPLC) | Bio-Techne GmbH, Germany | 30 μg/mouse |
| Alexa Fluor® 488 anti-mouse Ly-6G antibody | 1A8 | BioLegend, Fell, Germany | 3 μg/mouse |
| Cromolyn sodium salt | assay ≥ 95 % | Sigma Aldrich, Taufkirchen, Germany | 0.2 μg/g (BW) |
| Dyllight 649 platelet labeling | X649 | Emfret | 3 μg/mouse |
| InVivoMAb anti-mouse LFA-1α (CD11a) | M17/4 | Bio X Cell, Lebanon, USA | 30 μg/mouse |
| InVivoMAb anti-mouse/human CD11b | M1/70 | Bio X Cell, Lebanon, USA | 30 μg/mouse |
| InVivoMAb anti-mouse E-selectin | 9A9 | Bio X Cell, Lebanon, USA | 20 μg/mouse |
| InVivoMAbs rat IgG1 isotype control | HRPN | Bio X Cell, Lebanon, USA | 30 μg/mouse |
| InVivoMAbs rat IgG2a isotype control | 2A3 | Bio X Cell, Lebanon, USA | 30 μg/mouse |
| In vivo MAbs rat IgG2a isotype control | 2A3 | Bio X Cell, Lebanon, USA | 30 μg/mouse |
| Purified anti-mouse TNF-α antibody | MP6-XT22 | BioLegend, Fell, Germany | 30 μg/mouse |
| Purified rat anti-mouse CD62P antibody | RB40.34 | BD Pharmingen, Germany | 20 μg/mouse |
| Ultra-LEAFTM purified Rat IgG2b, κ Isotype Ctrl antibody | RTK4530 | BioLegend, Fell, Germany | 20 μg/mouse |
